# Critical thinking dispositions and perceived support-dependence discrepancy in GenAI-assisted learning: evidence from human–AI interaction pathways

**DOI:** 10.3389/fpsyg.2026.1913577

**Published:** 2026-10-28

**Authors:** Xiaoli Wang, Tianpei Xu, Xianghong Meng

**Affiliations:** 1 College of Educational Science, Inner Mongolia Minzu University, Tongliao, China; 2 College of Education, Hulunbuir University, Hulunbuir, China

**Keywords:** cognitive enhancement, cognitive offloading, critical thinking dispositions, generative artificial intelligence, perceived support-dependence discrepancy

## Abstract

**Background:**

Generative artificial intelligence (GenAI) can support academic performance while also creating a perceived gap between AI-supported task completion and independently available competence. This study examined how multidimensional critical thinking dispositions are associated with cognitive offloading, cognitive enhancement, and perceived support-dependence discrepancy (PSD) in GenAI-assisted learning.

**Methods:**

A cross-sectional survey was administered through Wenjuanxing from March 15 to May 20, 2026, using stratified purposive sampling across six Chinese universities. Of 990 submitted questionnaires, 797 eligible and complete responses were retained. The hypothesized pathways were tested using structural equation modeling, with a separate observed-score moderation analysis for academic stress and additional robustness and measurement-model assessments.

**Results:**

Inquisitiveness was positively associated with cognitive enhancement (β = 0.515, *p* < 0.001) and cognitive offloading (β = 0.270, *p* < 0.001). Open-mindedness (β = 0.091, *p* = 0.039) and systematicity (β = 0.096, *p* = 0.029) showed small positive associations with cognitive enhancement, whereas analyticity and truth-seeking were not significantly associated with either interaction pathway. Cognitive offloading (β = 0.448, *p* < 0.001) and cognitive enhancement (β = 0.207, *p* < 0.001) were positively associated with PSD. The Academic Stress × Cognitive Offloading interaction was not significant (β = 0.048, *p* = 0.123; delta R-squared = 0.002). Alternative CFA specifications, a common-method-factor analysis, and reliability sensitivity analyses yielded broadly consistent focal conclusions, while the HTMT assessment provided qualified evidence of discriminant validity.

**Conclusion:**

Critical thinking dispositions showed heterogeneous associations with GenAI interaction pathways, and both offloading and enhancement were associated with perceived support-dependent competence gaps. Because the outcome was measured with a single self-report item and the design was cross-sectional, the findings indicate statistical associations rather than objectively verified overconfidence, calibration bias, or causal effects.

## 1 Introduction

### 1.1 Research background

In digitally mediated learning environments, GenAI is increasingly used for information retrieval, explanation, drafting, revision, and problem solving (Chan and Hu, 2023; Lodge et al., 2023; Wang, 2025). Students nevertheless differ in how they use this support. Some retain control over task goals, prompt formulation, output evaluation, and revision and use GenAI to elaborate or challenge their own reasoning, whereas others rely more heavily on generated responses and delegate parts of information organization or reasoning to the system (Kim et al., 2026; Lee et al., 2025). These patterns can be described as cognitive enhancement and cognitive offloading, respectively. They are treated here as related tendencies that may co-occur rather than as mutually exclusive learner types, consistent with distributed accounts of cognition in human–tool systems (Clark, 2025; Hollan et al., 2000).

### 1.2 Research questions

Recent research has moved beyond simple adoption intention by documenting heterogeneous student–GenAI interaction patterns, strategic prompting, and learner–AI interactivity (Kim et al., 2026; Lee and Palmer, 2025; Zamfirescu-Pereira et al., 2023). Nevertheless, three connected gaps remain. First, much of the educational GenAI literature continues to emphasize adoption, perceived usefulness, use frequency, or system and task characteristics (Chan and Hu, 2023; Sergeeva et al., 2025; Strzelecki, 2024), whereas comparatively less evidence identifies learner-level dispositions associated with cognitively substitutive versus cognitively constructive interaction. Second, research on AI-supported performance and metacognitive monitoring has only recently begun to examine whether learners can distinguish supported task success from independently reproducible competence (Fernandes et al., 2026; Tankelevitch et al., 2024). Third, few studies integrate multidimensional critical thinking dispositions, distinct interaction pathways, a cautiously defined support-dependence outcome, and contextual demands within one empirically testable model.

Because GenAI can generate fluent and apparently coherent text with relatively low user effort, learners must decide whether to verify, challenge, reorganize, or accept the output (Farquhar et al., 2024; Lodge et al., 2023). Critical thinking dispositions describe motivational tendencies toward inquiry, evidence evaluation, open-minded judgment, and systematic reasoning (Facione, 2000; Facione et al., 1995) and may therefore be associated with how learners distribute cognitive responsibility between themselves and the system. Recent evidence that GenAI use can alter perceived cognitive effort and critical evaluation further supports examining these associations separately for cognitive offloading and cognitive enhancement (Essien et al., 2024; Lee et al., 2025).

These interaction patterns may also be relevant to metacognitive monitoring, defined in this study as learners' ongoing judgments of what they understand, know, or can reproduce independently, rather than retrospective judgments about the effectiveness of the strategy used (Koriat and Bjork, 2005; Nelson, 1990). In GenAI-assisted learning, this monitoring process concerns whether learners understand an AI-generated explanation and can reproduce its reasoning without support. Accordingly, the present study treats the distinction between supported performance and independently available competence as a monitoring-related issue rather than as a complete operationalization of metacognitive monitoring. Fluent responses can reduce the salience of unresolved conflict and increase reliance on accessibility or processing fluency when learners judge what they know (Koriat and Bjork, 2005; Nelson, 1990). Research on Internet search and GenAI-assisted performance suggests that externally supported success may complicate judgments about what knowledge or competence is independently available (Fernandes et al., 2026; Fisher et al., 2015; Ward, 2021). The single outcome item used in this study captures a perceived contrast between AI-supported task completion and unsupported independent competence. The outcome is therefore interpreted as perceived support-dependence discrepancy, namely, a monitoring-related perception of the gap between AI-supported performance and unaided competence, rather than as a direct assessment of metacognitive monitoring or as evidence of overconfidence, cognitive-ownership confusion, or objective calibration bias. Distributed-cognition and source-monitoring perspectives are used only to frame why such perceived contrasts may arise in human–AI learning contexts, not to claim that cognitive ownership was empirically misattributed (Hollan et al., 2000; Tankelevitch et al., 2024).

Accordingly, this study addresses the following research questions:

RQ1: How are five dimensions of critical thinking disposition statistically associated with cognitive offloading and cognitive enhancement in GenAI-assisted academic learning?

RQ2: Are critical thinking dispositions indirectly associated with perceived support-dependence discrepancy through cognitive offloading and cognitive enhancement?

RQ3: Does academic stress moderate the association between cognitive offloading and perceived support-dependence discrepancy?

### 1.3 Research contributions

To address the identified gaps, this study develops and tests a multipath model linking five critical thinking dispositions to cognitive offloading, cognitive enhancement, and a cautiously defined perceived AI/no-AI support-dependence discrepancy. The contributions are empirical and theory-building, but they do not establish causal effects or an objectively measured cognitive-ownership mechanism.

First, the study separates cognitive offloading and cognitive enhancement rather than treating GenAI use as a single adoption outcome. It examines critical thinking dispositions as learner-level antecedents and estimates their dimension-specific statistical associations with the two interaction pathways. This provides an empirically testable account of heterogeneity in cognitive agency during GenAI-assisted learning.

Second, the study links both interaction pathways to perceived support-dependence discrepancy. The positive associations indicate that both substitutive and constructive forms of GenAI engagement may be accompanied by a stronger perceived gap between AI-supported performance and unaided independent competence. This interpretation does not assume that learners are necessarily overconfident or confused about cognitive ownership; rather, it treats PSD as a self-reported awareness of support-dependent competence that requires further validation with behavioral and performance-based measures.

Third, the study tests a specific contextual moderation hypothesis. Academic stress showed a small positive main association with perceived support-dependence discrepancy, whereas the Academic Stress × Cognitive Offloading interaction was not statistically significant. This result indicates that the hypothesized moderation was not detected in the present sample; it does not establish invariance across stress levels, institutions, or learning tasks.

## 2 Literature review and hypothesis development

### 2.1 Cognitive offloading and cognitive enhancement in GenAI-assisted learning

GenAI-assisted learning may involve cognitive offloading and cognitive enhancement. Cognitive offloading refers to using external tools to reduce or replace internally performed cognitive operations (Risko and Gilbert, 2016), whereas cognitive enhancement refers here to constructive and interactive engagement in which learners retain task control and use GenAI for elaboration, comparison, verification, and reconstruction (Chi and Wylie, 2014; Clark, 2025). The constructs are modeled as correlated latent tendencies. They were empirically distinguishable in the present measurement model, including an offloading–enhancement HTMT ratio of 0.654 with a 95% bootstrap confidence interval of [0.610, 0.695], but they are not assumed to be mutually exclusive or opposite endpoints of a single continuum.

Cognitive offloading can be adaptive when external tools preserve limited working resources for higher-order operations (Ball et al., 2022; Risko and Gilbert, 2016), but it can become substitutive when learners delegate the central reasoning required for later independent performance or knowledge retention (Abbas et al., 2024; Barcaui, 2025). Cognitive enhancement, in contrast, requires constructive or interactive engagement in which learners question outputs, compare sources, generate counterarguments, and integrate information into existing knowledge structures (Chi and Wylie, 2014; de la Puente et al., 2024; Suriano et al., 2025). The key distinction is therefore not whether GenAI is used, but which cognitive operations remain under learner control and can later be reproduced without support.

Although prior work has described heterogeneous interaction patterns, these patterns are often explained through task complexity, prompt design, scaffolding, or system features (Kim et al., 2026; Lee and Palmer, 2025; Yan et al., 2025; Zamfirescu-Pereira et al., 2023). Less evidence identifies learner-level dispositions associated with offloading and enhancement, and still less work connects these pathways to judgments of independent competence after AI support is removed. The present model therefore integrates disposition-specific antecedents, two correlated interaction pathways, a perceived support-dependence outcome, and a prespecified contextual moderator, while recognizing that task type and complexity remain potential sources of heterogeneity. Recent AI-literacy frameworks and validated GenAI-use instruments commonly assess knowledge of AI, operational competence, ethical awareness, critical evaluation, general reliance, or patterns of technology use. These domains are related to, but not interchangeable with, the context-specific cognitive offloading and enhancement tendencies examined in the present study. Accordingly, the current measures are treated as preliminary contextualized operationalizations, and future research should evaluate their convergent and incremental validity against established AI-literacy and GenAI-use measures.

### 2.2 Critical thinking dispositions and heterogeneity in interaction pathways

This study examines critical thinking disposition as an antecedent of learner–AI interaction patterns. Drawing on Facione's framework and the CCTDI tradition, it focuses on inquisitiveness, analyticity, truth-seeking, open-mindedness, and systematicity (Facione, 2000; Facione et al., 1995, 2001). These dimensions represent motivational tendencies toward inquiry, logical evaluation, evidence seeking, consideration of alternative views, and structured reasoning. Prior research has more often examined critical thinking as an outcome of AI-supported instruction than as a learner characteristic associated with how GenAI is used (de la Puente et al., 2024; Essien et al., 2024; Liang and Wu, 2024; Yusuf et al., 2024); the antecedent role of specific dispositions therefore remains comparatively underexplored.

First, disposition-specific associations with cognitive offloading and cognitive enhancement remain insufficiently tested. Inquisitiveness reflects sustained interest in unresolved questions and may increase the breadth of GenAI engagement. Analyticity and truth-seeking emphasize logical adequacy, evidence quality, and source verification and may be expressed as checking behavior when uncertainty cues are salient (Facione, 2000; Pennycook and Rand, 2019). Open-mindedness supports consideration of alternative explanations, whereas systematicity supports the organization and integration of fragmented information (Facione et al., 1995; Stanovich and West, 1997). These tendencies may be associated with different forms of offloading and enhancement, but their direction and relative strength require empirical examination without assuming that any disposition automatically produces a particular interaction behavior.

Second, the downstream correlates of different interaction pathways that are relevant to metacognitive monitoring remain insufficiently specified. Reliance on external information tools can complicate judgments of internally available knowledge, and GenAI introduces additional monitoring demands because supported performance may be difficult to separate from independent competence (Fernandes et al., 2026; Fisher et al., 2015; Tankelevitch et al., 2024; Ward, 2021). Here, metacognitive monitoring refers to learners' ongoing judgments about their current understanding and independently reproducible competence. Academic stress can alter learning strategies and impair metacognitive monitoring (Reyes et al., 2015; Trigueros et al., 2020; Vogel and Schwabe, 2016), but whether it moderates the offloading–support-dependence discrepancy association has not been established. The present study tests this specific conditional association while treating perceived support-dependence discrepancy as a self-reported, monitoring-related perception of the gap between AI-supported and unaided competence. This construct is not equated with metacognitive monitoring itself, and cognitive-ownership misattribution is not treated as a measured mechanism.

### 2.3 Hypothesis development and theoretical model

The depth-of-processing perspective explains how learner dispositions may be associated with different forms of GenAI engagement. Durable learning depends more strongly on semantic elaboration, organization, comparison, and integration than on surface familiarity or fluent exposure (Craik and Lockhart, 1972; Pintrich, 1999). In GenAI-assisted learning, enhancement is therefore characterized by learner-generated questions, verification, and reconstruction of system output, whereas substitutive offloading reduces opportunities for the central processing required for later independent performance. Distributed cognition explains a different part of the model: a successful AI-assisted product is produced by a coupled learner–tool system rather than by the learner alone (Clark, 2025; Hollan et al., 2000). Consequently, learners' perceived support-dependence may depend on whether they can identify which cognitive operations were independently generated and which were enabled by external AI support. The two perspectives thus explain distinct links in the model: depth of processing informs the interaction pathways, whereas distributed cognition informs why learners may perceive a gap between supported task completion and unsupported independent performance.

#### 2.3.1 Disposition-specific associations with human–AI interaction pathways

Inquisitiveness reflects a motivational tendency to pursue explanations and unresolved questions (Facione, 2000). It is expected to be associated with cognitive enhancement when learners use follow-up prompts, request alternatives, compare explanations, and continue inquiry beyond the first response, a pattern consistent with constructive and interactive engagement (Chi and Wylie, 2014; Suriano et al., 2025). Its association with cognitive offloading is more tentative and requires a different theoretical rationale. In unstructured GenAI-supported learning, highly inquisitive learners may expand the scope and complexity of the questions they pursue. When this expanded inquiry exceeds their available time, prior knowledge, or processing resources, GenAI may be used not only for exploration but also for preliminary retrieval, organization, and response structuring. In this sense, inquisitiveness may be linked to offloading through high-intensity, exploratory tool engagement rather than through an intrinsic tendency toward uncritical dependence. The present study therefore treats the inquisitiveness–offloading path as a cautious empirical hypothesis, not as evidence that inquisitive learners deliberately or optimally allocate cognitive subtasks.

Analyticity and truth-seeking emphasize logical adequacy, evidence quality, and source verification (Facione, 2000; Pennycook and Rand, 2019). These dispositions may reduce uncritical adoption and support enhancement when task cues make uncertainty, contradiction, or verification needs salient. In terms of automation trust, monitoring is more likely to be activated when users perceive error risk or conflicting evidence; highly fluent output may weaken these cues and permit continued reliance despite strong analytic dispositions (Farquhar et al., 2024; Hoff and Bashir, 2015). Metacognitive monitoring is therefore proposed as a possible triggering process that translates disposition into observable checking or reconstruction behavior; it was not measured as a separate mediator in the present study.

Open-mindedness and systematicity are expected to be more directly related to cognitive enhancement. Open-mindedness supports the consideration of alternative explanations and revision of prior beliefs when evidence conflicts with an initial position, whereas systematicity supports the orderly organization of fragmented claims, evidence, and conclusions (Facione et al., 1995; Stanovich and West, 1997). These tendencies are compatible with comparing multiple GenAI outputs and reconstructing them within an existing knowledge framework. Their proposed positive associations with offloading are more tentative: greater engagement with multiple perspectives and structured task completion may increase overall tool use without necessarily implying uncritical dependence.

Integrating these theoretical perspectives, and recognizing that different dimensions of critical thinking disposition may produce distinct resource-allocation patterns within a distributed human–AI cognitive system, we propose the following hypotheses:

H1: The dimensions of critical thinking disposition show differential associations with cognitive offloading.

H1a: Inquisitiveness is associated with cognitive offloading, with the direction of this association treated as an empirical question in the present GenAI-supported learning context.

H1b: Analyticity is negatively associated with cognitive offloading.

H1c: Truth-seeking is negatively associated with cognitive offloading.

H1d: Open-mindedness is positively associated with cognitive offloading.

H1e: Systematicity is positively associated with cognitive offloading.

H2: The dimensions of critical thinking disposition show differential associations with cognitive enhancement.

H2a: Inquisitiveness is positively associated with cognitive enhancement.

H2b: Analyticity is positively associated with cognitive enhancement.

H2c: Truth-seeking is positively associated with cognitive enhancement.

H2d: Open-mindedness is positively associated with cognitive enhancement.

H2e: Systematicity is positively associated with cognitive enhancement.

#### 2.3.2 Formation of perceived support-dependence discrepancy

The outcome item used in this study captures learners' perceived contrast between what they can accomplish with GenAI support and what they can accomplish independently without such support. A higher score therefore indicates stronger perceived support-dependence rather than necessarily indicating overconfidence, blind reliance, or objective calibration error. Cognitive offloading may be positively associated with this discrepancy because substitutive reliance on GenAI can reduce opportunities for independent practice while making unaided task demands more difficult when support is removed. Cognitive enhancement may also be positively associated with this discrepancy for a different reason: learners who actively compare, verify, and reconstruct AI-generated content may become more aware of the limits of their unaided competence. Thus, positive associations with PSD are interpreted as recognized support-dependent competence gaps rather than as direct evidence of illusory self-assessment. Cognitive ownership and metacognitive calibration were not directly measured and are not treated as established mechanisms in the present study. Accordingly, we propose the following associations:

H3: Cognitive offloading is positively associated with perceived support-dependence discrepancy.

H4: Cognitive enhancement is positively associated with perceived support-dependence discrepancy.

#### 2.3.3 Contextual moderating effect of academic stress

Academic stress can alter strategy selection, learning behavior, and reliance on external support (Trigueros et al., 2020; Vogel and Schwabe, 2016). Under evaluative pressure, learners may adopt strategies that protect perceived self-worth or permit faster task completion (Covington, 1984). Stress has also been associated with reduced metacognitive accuracy (Reyes et al., 2015), and recent GenAI research suggests that academic stress can be related to AI dependence (Acosta-Enriquez et al., 2025). Higher perceived stress may therefore strengthen the association between cognitive offloading and perceived support-dependence discrepancy. The moderation test is restricted to the offloading pathway; no enhancement × stress interaction is hypothesized or tested.

H5: Academic stress positively moderates the association between cognitive offloading and perceived support-dependence discrepancy. Based on the proposed cognitive-processing mechanism, the distributed-cognition perspective, and hypotheses H1–H5, this study develops the conceptual framework shown in Figure 1.

**Figure 1:**
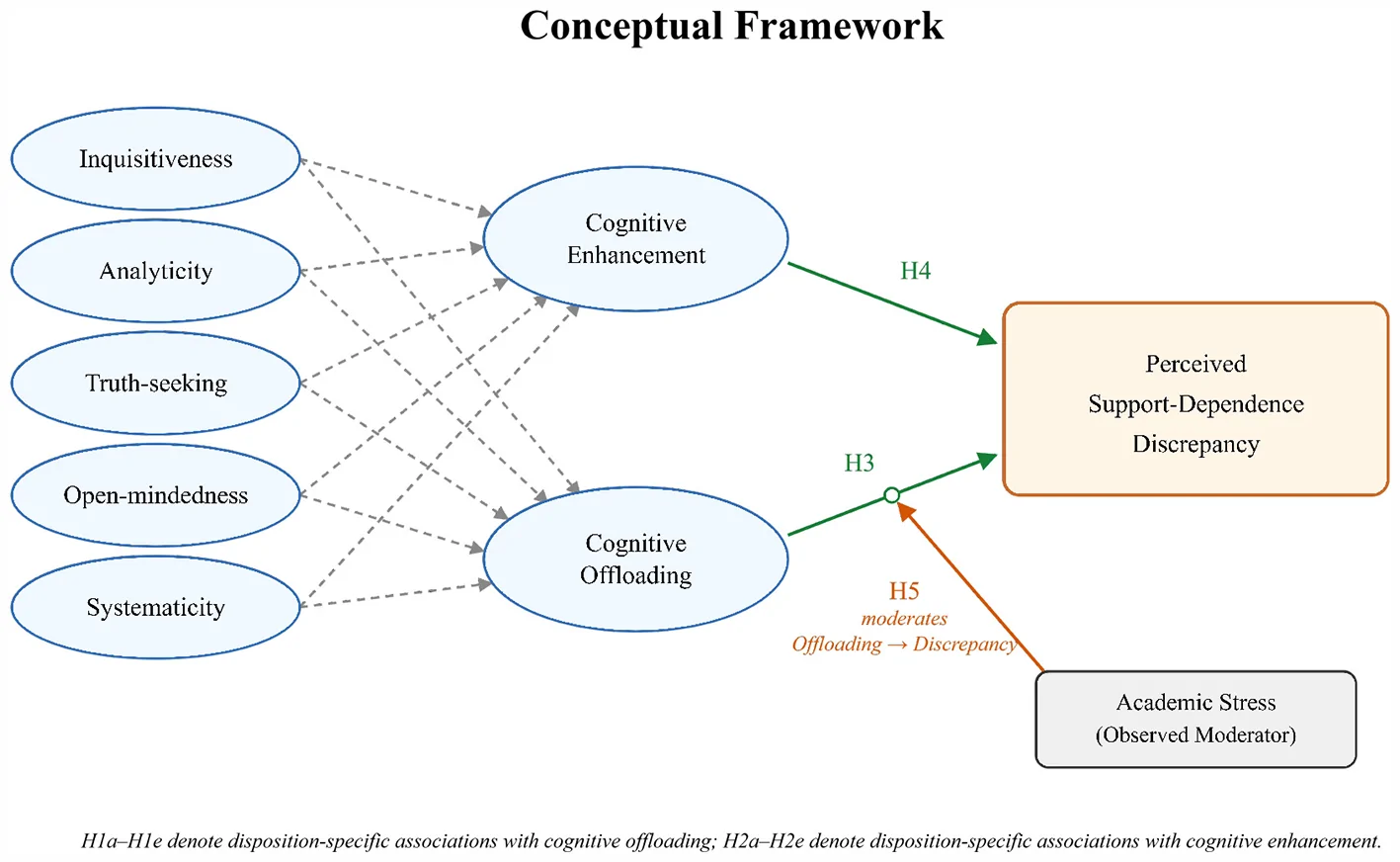
Conceptual model linking critical thinking dispositions, cognitive offloading, cognitive enhancement, perceived support-dependence discrepancy, and the moderating role of academic stress.

## 3 Methodology

### 3.1 Participants and data collection

The target population comprised university students in China who had previously used GenAI for academic learning. Data were collected from March 15 to May 20, 2026, through Wenjuanxing (WJX), a web-based survey platform used for online questionnaire administration in China. Stratified purposive sampling was organized through a six-cell region-by-institution-type frame: eastern, central, and western China × regular university and Double First-Class university. Six universities participated, with one regular university and one Double First-Class university in each region. After ethical approval, designated contacts at the six institutions distributed the anonymous survey link through student learning and communication networks. The survey instructions asked participants to answer with reference to their usual use of GenAI in academic learning activities, including coursework, assignments, academic writing, information retrieval, and problem solving, and not with reference to entertainment or other non-academic uses. The design therefore assessed cross-task tendencies rather than behavior within a single standardized task. The design ensured coverage of each sampling cell but was not probability-based and does not justify claims of proportional national representation.

A total of 990 questionnaires were submitted. Eligibility required voluntary consent, current university-student status, and prior GenAI use for coursework, assignments, academic writing, information retrieval, or other academic learning activities. Complete-case screening excluded submissions with missing focal data, failure on either of two Instructional Manipulation Checks, straight-line or otherwise patterned responding, or failure to meet the university-student or GenAI-use eligibility items. In total, 193 submissions were excluded and 797 were retained, corresponding to a valid-case proportion of 80.51% among submitted questionnaires. Because recruitment used a decentralized open link, an invitation-based response rate could not be calculated. The retained analysis archive preserved the final inclusion or exclusion status but did not retain mutually exclusive reason-specific exclusion counts. This was a data-retention limitation rather than a consequence of de-identification. Consequently, reason-specific exclusion counts could not be reconstructed, and no estimated values were substituted. Participant characteristics are summarized in Table 1.

**Table 1:** Demographic characteristics of participants (*N* = 797).

Variable	Category	*N*	%
Gender	Male	374	46.9
	Female	423	53.1
Grade level	Lower-division undergraduates (first-/second-year)	532	66.8
	Upper-division undergraduates (third-/fourth-year)	154	19.3
	Postgraduates	111	13.9
Institution type	Regular universities	671	84.2
	Double First-Class universities	126	15.8
Disciplinary background	STEM	245	30.7
	Humanities and social sciences	177	22.2
	Arts and sports	166	20.8
	Computer science and artificial intelligence	125	15.7
	Agriculture, forestry, and medicine	84	10.5

Percentages may not total 100% because of rounding.

### 3.2 Measurement instruments and variable operationalization

All multi-item constructs used five-point Likert-type response options ranging from 1 (strongly disagree) to 5 (strongly agree). The five critical-thinking disposition dimensions were adapted from the California Critical Thinking Disposition Inventory developed by Facione and colleagues, with reference to the Chinese validation reported by Facione et al. (2001); Peng et al. (2004). Cognitive offloading was operationalized through three context-specific items developed from the theoretical definition of externalized cognitive processing proposed by Risko and Gilbert (2016) and informed by subsequent offloading research (Ball et al., 2022). Cognitive enhancement was operationalized through three context-specific items informed by distributed cognition and the constructive and interactive engagement processes described in the ICAP framework (Chi and Wylie, 2014; Hollan et al., 2000). The offloading and enhancement items were therefore contextualized measures developed for GenAI-assisted academic learning rather than previously validated generic GenAI-use scales. The administered Chinese items were developed through translation, independent back-translation, and item-by-item reconciliation. Because the original WJX export was not retained, Supplementary Appendix A presents an English reporting reconstruction aligned with the analysis archive and manuscript documentation rather than a verbatim export of the administered questionnaire. The appendix also reports the screening questions, attention checks, response scales, and variable coding used in the analysis.

#### 3.2.1 Human–AI interaction patterns

Two three-item latent constructs represented human–AI interaction tendencies. Cognitive offloading assessed reliance on AI-generated output as a substitute for parts of the learner's own processing, consistent with the theoretical definition of externalized cognitive processing (Risko and Gilbert, 2016). Cognitive enhancement assessed questioning, comparison, verification, and reconstruction while the learner retained task control, consistent with constructive and interactive engagement in the ICAP framework (Chi and Wylie, 2014). Because the measures were contextualized for the present study and were not validated in an independent pilot sample, they are interpreted as preliminary, context-specific operationalizations rather than established generic GenAI-use scales.

#### 3.2.2 Critical thinking dispositions

To measure higher-order cognitive dispositions, five dimensions were adapted from the California Critical Thinking Disposition Inventory developed by Facione, Facione, and Giancarlo: inquisitiveness, analyticity, truth-seeking, open-mindedness, and systematicity (Facione et al., 2001). Item adaptation also referred to the conceptual definitions in Facione (2000) and the Chinese validation reported by Peng et al. (2004). The combined adapted items demonstrated high internal consistency in the present sample (overall Cronbach's α = 0.92).

#### 3.2.3 Contextual moderator and perceived support-dependence discrepancy

Academic stress and perceived support-dependence discrepancy were modeled as single-item observed variables, and both used five-point response scales. Academic stress was rated from 1 (very low) to 5 (very high). Perceived support-dependence discrepancy was rated from 1 (strongly disagree) to 5 (strongly agree) using the statement: “When I use AI to solve problems, I often feel that my ability to solve problems independently has improved; however, when I face similar tasks without AI, I find that I still cannot complete them independently.” The item captures a self-reported awareness of the contrast between AI-supported problem solving and unsupported independent task completion. It does not compare a confidence judgment with an objectively scored unaided performance task and therefore is not interpreted as a direct measure of overconfidence, cognitive-ownership confusion, or objective calibration bias.

### 3.3 Data analysis strategy

SPSS 26.0 and AMOS 24.0 were used. Descriptive statistics and Pearson correlations were calculated first. Confirmatory factor analysis evaluated the seven multi-item constructs, and discriminant validity was assessed using the Fornell–Larcker criterion and the heterotrait–monotrait ratio (HTMT) with 5,000 bootstrap confidence intervals (Fornell and Larcker, 1981; Henseler et al., 2015). Four measurement specifications were compared: seven correlated first-order factors; a higher-order model in which the five critical-thinking dimensions loaded on a second-order critical-thinking factor; a merged-dimension model in which the indicators of analyticity, truth-seeking, and open-mindedness loaded on a single factor while the remaining constructs were retained as separate correlated factors; and a single-factor model in which all 21 multi-item indicators loaded on one common factor. Model fit was evaluated using χ^2^/*df*, CFI, TLI, RMSEA with its 90% confidence interval, and SRMR (Hu and Bentler, 1999). The latent structural model was estimated with maximum likelihood. Standardized coefficients, standard errors, normal-theory 95% confidence intervals, *p* values, and squared multiple correlations were reported for direct paths, whereas indirect associations were evaluated using 5,000 bias-corrected bootstrap resamples. Moderation was examined separately using observed composite scores: the three offloading items and the three enhancement items were averaged, offloading and academic stress were mean-centered, and the Academic Stress × Cognitive Offloading product term was added in a second hierarchical-regression step. Gender, grade level, institution type, disciplinary background, and GenAI-use frequency were examined in an auxiliary robustness model.

Common-method risk was addressed procedurally through anonymous participation, task-domain instructions, separated questionnaire blocks, eligibility screening, and two attention checks (Podsakoff et al., 2003). Harman's single-factor result (38.12% for the first factor) was retained only as a descriptive diagnostic and was not interpreted as proof that common-method variance was absent. A common latent method factor was added to the structural specification; for identification, it was specified as orthogonal to the substantive factors, its variance was fixed at 1.00, and its indicator loadings were freely estimated. Model fit changed from χ^2^/*df* = 2.34, CFI = 0.945, TLI = 0.932, RMSEA = 0.041, and SRMR = 0.038 to χ^2^/*df* = 2.21, CFI = 0.951, TLI = 0.939, RMSEA = 0.039, and SRMR = 0.035. The CFI improvement was small (ΔCFI = 0.006), item-loading changes ranged from 0.018 to 0.074, and focal-path changes ranged from 0.006 to 0.036. These results do not eliminate common-method bias, but they indicate that a general method factor did not materially alter the focal associations.

### 3.4 Measurement model evaluation

The hypothesized seven-factor measurement model showed acceptable fit: χ^2^ = 356.42, *df* = 168, χ^2^/*df* = 2.12, CFI = 0.954, TLI = 0.943, RMSEA = 0.038, 90% CI [0.033, 0.043], and SRMR = 0.035. Standardized factor loadings ranged from 0.602 to 0.818, and Cronbach's α and composite reliability exceeded 0.70 for all multi-item constructs. AVE was below 0.50 for cognitive offloading (0.495), cognitive enhancement (0.478), and open-mindedness (0.442). Adequate composite reliability partially mitigates marginal AVE values, but it does not remove the convergent-validity concern. Construct-level reliability and validity results are reported in Table 2.

**Table 2:** Measurement model reliability, validity, and sample items.

Constructs	Sample item	Factor loadings	Cronbach's α	CR	AVE
Cognitive offloading (*n* = 3)	If the AI's response meets the basic requirements of the assignment, I adopt it directly without further modification.	0.617 - 0.756	0.737	0.745	0.495
Cognitive enhancement (*n* = 3)	I frequently input my perspectives into the AI, prompting it to generate counterarguments or identify logical flaws in my reasoning.	0.648 - 0.726	0.731	0.733	0.478
Inquisitiveness (*n* = 3)	When faced with complex problems, I have a strong urge to explore the underlying mechanisms behind them.	0.633 - 0.760	0.754	0.758	0.512
Analyticity (*n* = 3)	When reading lengthy texts, I actively look for logical leaps or flawed causal reasoning.	0.717 - 0.811	0.804	0.806	0.581
Truth-seeking (*n* = 3)	Before citing data or literature provided by the internet or AI, I have the habit of verifying the original sources.	0.684 - 0.782	0.761	0.765	0.521
Open-mindedness (*n* = 3)	When conclusive evidence or AI reasoning contradicts my views, I rationally revise my perspective.	0.602 - 0.705	0.700	0.703	0.442
Systematicity (*n* = 3)	When confronted with fragmented information, I instinctively reorganize it to construct a clear framework.	0.752 - 0.818	0.834	0.835	0.628

α, Cronbach's α; CR, composite reliability; AVE, average variance extracted. All standardized factor loadings were significant at *p* < 0.001.

As shown in Table 3, the square root of AVE for most latent variables exceeded their Pearson correlations with other variables. The only exception was open-mindedness: its AVE square root was 0.665, slightly lower than its correlations with truth-seeking (0.674) and systematicity (0.685).

**Table 3:** Discriminant validity and correlation matrix.

Construct	*M*	*SD*	1	2	3	4	5	6	7	8	9
1. Cognitive Offloading	3.12	0.84	**0.704**								
2. Cognitive Enhancement	3.30	0.79	0.525	**0.691**							
3. Inquisitiveness	3.36	0.76	0.366	0.577	**0.716**						
4. Analyticity	3.49	0.75	0.292	0.364	0.538	**0.762**					
5. Truth-seeking	3.56	0.72	0.308	0.366	0.469	0.708	**0.722**				
6. Open-mindedness	3.57	0.66	0.290	0.354	0.404	0.637	0.674	**0.665**			
7. Systematicity	3.60	0.74	0.287	0.353	0.408	0.671	0.643	0.685	**0.792**		
8. PSD (Support-Dependence)	2.83	1.10	0.556	0.442	0.298	0.235	0.231	0.248	0.247	**–**	
9. Academic Stress	3.28	0.97	0.270	0.307	0.242	0.089	0.105	0.124	0.067	0.218	**–**

Bold diagonal values are the square roots of AVE; off-diagonal values are Pearson correlation coefficients. AVE is not reported for the single-item measures of perceived support-dependence discrepancy and academic stress; their diagonal entries are therefore omitted. PSD, perceived support-dependence discrepancy.

The Fornell–Larcker comparison was satisfied for most constructs, with open-mindedness remaining the exception. HTMT values ranged from 0.339 to 0.887. The largest ratios were observed for open-mindedness and systematicity (HTMT = 0.887, 95% bootstrap CI [0.851, 0.916]), open-mindedness and truth-seeking (HTMT = 0.861, 95% CI [0.823, 0.892]), and analyticity and truth-seeking (HTMT = 0.853, 95% CI [0.817, 0.884]). None of the bootstrap intervals included 1.00. The combined evidence provided qualified support for empirical separability, while the high HTMT values and the Fornell–Larcker exception indicated substantial overlap among several critical-thinking dimensions. The complete HTMT matrix and key bootstrap confidence intervals are reported in Supplementary Table S2.

Four measurement specifications were compared (Table 4 and Supplementary Table S1). The seven-correlated-factor model showed the best absolute fit and the lowest AIC and BIC. The higher-order critical-thinking model also showed acceptable fit, whereas the merged-dimension and single-factor models performed substantially worse. The evidence therefore favored retaining seven correlated first-order factors for the focal dimension-specific analysis, while the acceptable higher-order model indicated that the five critical-thinking dimensions share substantial common variance.

**Table 4:** Comparison of fit indices for alternative CFA models.

Model	χ^2^	*df*	χ^2^/*df*	CFI	TLI	RMSEA (90% CI)	SRMR	AIC	BIC
Seven correlated factors	356.42	168	2.12	0.954	0.943	0.038 [0.033, 0.043]	0.035	482.42	777.31
Higher-order CT	397.85	173	2.30	0.945	0.935	0.040 [0.035, 0.045]	0.040	513.85	785.34
Merged analyticity-truth-seeking-open-mindedness factor model	561.73	176	3.19	0.907	0.890	0.052 [0.047, 0.057]	0.064	671.73	929.18
Single factor	1438.26	189	7.61	0.699	0.663	0.091 [0.087, 0.096]	0.102	1,522.26	1,718.86

## 4 Data analysis and results

### 4.1 Descriptive statistics and correlation analysis

Descriptive statistics and the Pearson correlation matrix for the main study variables are reported in Table 3. To display the overall structure of associations among these variables, the correlation matrix is visualized as a heatmap in Figure 2.

**Figure 2:**
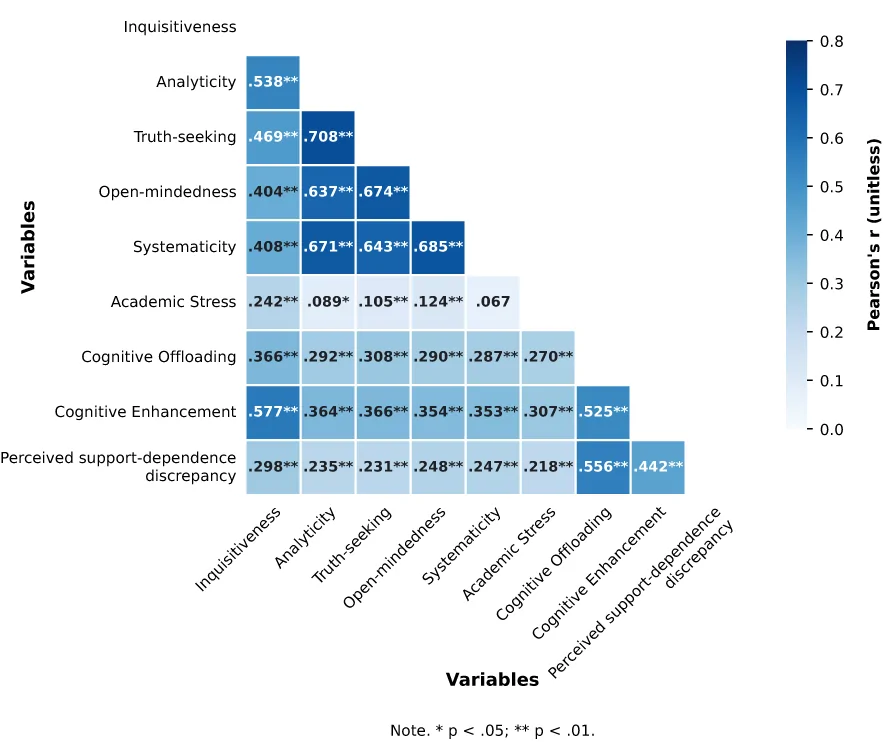
Correlation heatmap of the main study variables.

As shown in Figure 2, the critical-thinking dimensions were positively correlated with cognitive enhancement, cognitive offloading, and perceived support-dependence discrepancy. The correlations were descriptive and did not establish the direction of the structural paths. The pattern did not indicate severe bivariate multicollinearity and provided a basis for estimating the prespecified structural model.

### 4.2 Latent structural model and hypothesis testing

#### 4.2.1 Model fit assessment and correction for measurement error

Before testing the hypothesized structural paths, the overall fit of the structural equation model was evaluated using maximum likelihood estimation (MLE).

For the single-item perceived support-dependence discrepancy indicator, following established single-indicator SEM practice (Hayduk, 1987), the residual variance was fixed as:


$$\begin{array}{c} \theta_{\varepsilon }=\left(1-\rho \right)s_{y}^{2}, \end{array}$$ (1)

where ρ denotes the assumed reliability and $s_{y}^{2}$ denotes the observed variance. Because internal consistency cannot be estimated for a single item, 0.85 was treated as a modeling assumption rather than as an observed reliability coefficient. The latent structural model was re-estimated using assumed reliabilities of 0.70, 0.80, 0.85, 0.90, and 1.00. Across these specifications, the direction and significance classification of the focal latent paths remained unchanged. However, this sensitivity analysis does not substitute for a validated multi-item measure of support-dependence awareness or for objective measures of unaided performance. The observed-score moderation model was not included in this measurement-error correction series. Complete sensitivity estimates are reported in Supplementary Table S3. This sensitivity analysis does not substitute for a validated multi-item measure of support-dependence awareness or for objective measures of unaided performance.

The latent structural model showed acceptable fit: χ^2^/*df* = 2.34, RMSEA = 0.041, SRMR = 0.038, CFI = 0.945, and TLI = 0.932. These indices indicate that the specified covariance structure was compatible with the observed data; they do not establish causal direction or validate the two single-item indicators.

#### 4.2.2 Structural path analysis and hypothesis testing

The squared multiple correlations from AMOS indicated that the disposition predictors explained 16.7% of the variance in cognitive offloading and 35.6% of the variance in cognitive enhancement. Cognitive offloading and cognitive enhancement together accounted for 34.0% of the variance in perceived support-dependence discrepancy in the latent structural model.

Inquisitiveness was positively associated with cognitive enhancement (β = 0.515, *t* = 15.034, *p* < 0.001) and cognitive offloading (β = 0.270, *t* = 6.920, *p* < 0.001), supporting H1a and H2a. Open-mindedness (β = 0.091, *t* = 2.071, *p* = 0.039) and systematicity (β = 0.096, *t* = 2.185, *p* = 0.029) showed small positive associations with cognitive enhancement, supporting H2d and H2e. Analyticity and truth-seeking were not significantly associated with either interaction pathway, and open-mindedness and systematicity were not significantly associated with cognitive offloading. H1b, H1c, H1d, H1e, H2b, and H2c were therefore not supported.

Cognitive offloading (β = 0.448, *p* < 0.001) and cognitive enhancement (β = 0.207, *p* < 0.001) were positively associated with perceived support-dependence discrepancy, supporting H3 and H4. These coefficients indicate stronger self-reported gaps between AI-supported and unaided competence within the specified cross-sectional model; they should not be interpreted as causal effects or as evidence of objectively verified overconfidence or calibration bias. The complete path estimates are reported in Table 5, and the corresponding structural and moderation diagrams are shown in Figure 3.

**Table 5:** Latent structural model and hypothesis-testing results.

Structural Paths	Std. β	S.E.	*t*-value	*p*-value	95% LLCI	95% ULCI	Conclusion
Antecedent paths: CT dispositions→AI interaction modes							
H1a: Inquisitiveness → Cognitive Offloading	0.270	0.039	6.920	< 0.001^***^	0.194	0.346	Supported
H1b: Analyticity → Cognitive Offloading	−0.022	0.053	−0.412	0.680	−0.126	0.082	Not supported
H1c: Truth-seeking → Cognitive Offloading	0.095	0.051	1.846	0.065	−0.005	0.195	Not supported
H1d: Open-mindedness → Cognitive Offloading	0.080	0.050	1.600	0.110	−0.018	0.178	Not supported
H1e: Systematicity → Cognitive Offloading	0.076	0.050	1.522	0.128	−0.022	0.174	Not supported
H2a: Inquisitiveness → Cognitive Enhancement	0.515	0.034	15.034	< 0.001^***^	0.448	0.582	Supported
H2b: Analyticity → Cognitive Enhancement	−0.072	0.047	-1.552	0.121	−0.164	0.020	Not supported
H2c: Truth-seeking → Cognitive Enhancement	0.053	0.045	1.163	0.245	−0.035	0.141	Not supported
H2d: Open-mindedness → Cognitive Enhancement	0.091	0.044	2.071	0.039^*^	0.005	0.177	Supported
H2e: Systematicity → Cognitive Enhancement	0.096	0.044	2.185	0.029^*^	0.010	0.182	Supported
Outcome paths							
H3: Cognitive Offloading → PSD (Support-Dependence)	0.448	0.034	13.216	< 0.001^***^	0.381	0.515	Supported
H4: Cognitive Enhancement → PSD (Support-Dependence)	0.207	0.034	6.111	< 0.001^***^	0.140	0.274	Supported

Standardized coefficients are reported. Approximate 95% confidence intervals for direct paths were calculated as β±1.96 × S.E. Bootstrap confidence intervals based on 5,000 bias-corrected resamples are reported only for indirect associations. CT = critical thinking. ^*^p < 0.05; ^***^p < 0.001.

**Figure 3:**
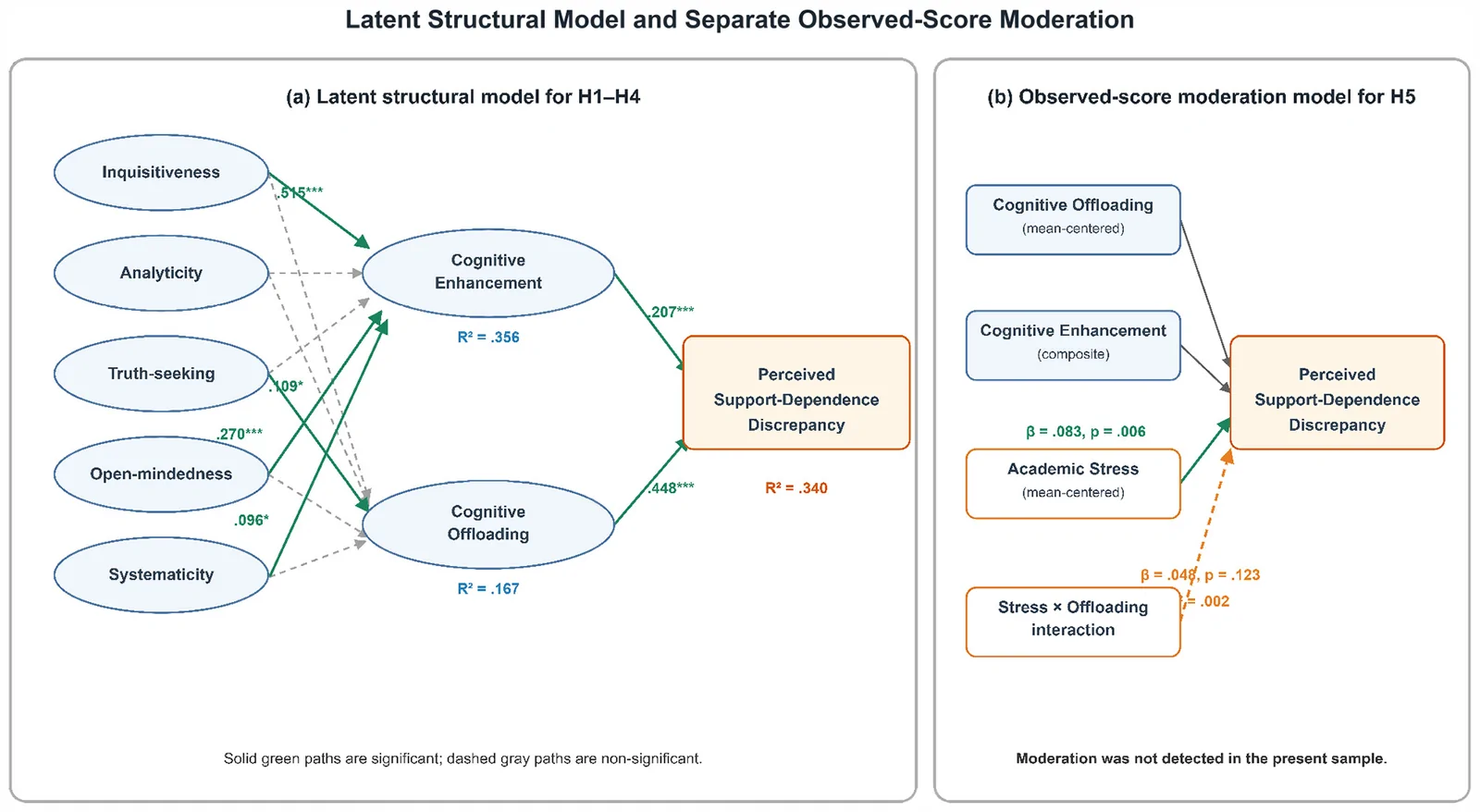
Latent structural model for H1–H4 and separate observed-score moderation model for H5. NCT, critical thinking. ^***^p < 0.001, ^*^p < 0.05. Covariances among the five exogenous variables were estimated but are omitted for visual clarity. Dashed lines indicate statistically nonsignificant paths.

### 4.3 Observed-score moderation analysis

Moderation was tested separately from the latent SEM using the mean-centered cognitive-offloading composite, the cognitive-enhancement composite, mean-centered academic stress, and their product term. In Step 1, the offloading, enhancement, and stress main effects were entered. In Step 2, the Academic Stress × Cognitive Offloading term was added. Academic stress showed a small positive main association with perceived support-dependence discrepancy (β = 0.083, *S*.*E*. = 0.030, 95% CI [0.024, 0.142], *p* = 0.006), whereas the interaction was not statistically significant (β = 0.048, *S*.*E*. = 0.031, 95% CI [-0.013, 0.109], *p* = 0.123). *R*^2^ increased from 0.338 to 0.340 (Δ*R*^2^ = 0.002). H5 was therefore not supported. The result indicates that moderation was not detected in the present sample; it does not establish invariance across stress levels. The complete observed-score moderation results are reported in Table 6.

**Table 6:** Observed-score moderation analysis.

Term	Std. β	S.E.	95% CI	*p*-value
Academic Stress	0.083	0.030	[0.024, 0.142]	0.006
Academic Stress × Cognitive Offloading	0.048	0.031	[-0.013, 0.109]	0.123
Model *R*^2^ before / after interaction	0.338 / 0.340	—	Δ*R*^2^ = 0.002	—

### 4.4 Bootstrap indirect associations

Bias-corrected bootstrap analysis with 5,000 resamples identified four indirect associations whose 95% confidence intervals did not include zero. Inquisitiveness was indirectly associated with perceived support-dependence discrepancy through cognitive offloading (indirect effect = 0.121, 95% CI [0.078, 0.174]) and cognitive enhancement (0.107, 95% CI [0.065, 0.153]). Open-mindedness (0.019, 95% CI [0.003, 0.041]) and systematicity (0.020, 95% CI [0.005, 0.043]) also showed small indirect associations through cognitive enhancement. These analyses test whether the interaction pathways transmit associations from dispositions to the perceived support-dependence outcome; they do not test objective calibration bias, cognitive-ownership processes, or temporal order. The complete bootstrap estimates are reported in Table 7.

**Table 7:** Bootstrap Estimates of indirect associations.

Indirect association path	Indirect effect	BootSE	BootLLCI	BootULCI	Result
Path 1: Inquisitiveness → Cognitive Offloading → PSD (Support-Dependence)	0.121	0.024	0.078	0.174	Significant
Path 2: Inquisitiveness → Cognitive Enhancement → PSD (Support-Dependence)	0.107	0.022	0.065	0.153	Significant
Path 3: Open-mindedness → Cognitive Enhancement → PSD (Support-Dependence)	0.019	0.009	0.003	0.041	Significant
Path 4: Systematicity → Cognitive Enhancement → PSD (Support-Dependence)	0.020	0.009	0.005	0.043	Significant

An indirect association is estimated as the product of the antecedent and subsequent path coefficients. A bias-corrected nonparametric percentile bootstrap with 5,000 resamples was used. LLCI and ULCI denote the lower and upper limits of the 95% confidence interval, respectively. An indirect association was considered statistically distinguishable from zero when its confidence interval did not include zero.

To compare the magnitudes and confidence intervals of the indirect effects across indirect pathways, a forest plot was generated (Figure 4). The 95% bootstrap confidence intervals for all four paths excluded zero, and all error bars lay to the right of the vertical reference line. The two paths involving inquisitiveness (Paths 1 and 2) had the largest indirect-effect estimates and stronger indirect associations with perceived support-dependence discrepancy than the paths involving open-mindedness and systematicity.

**Figure 4:**
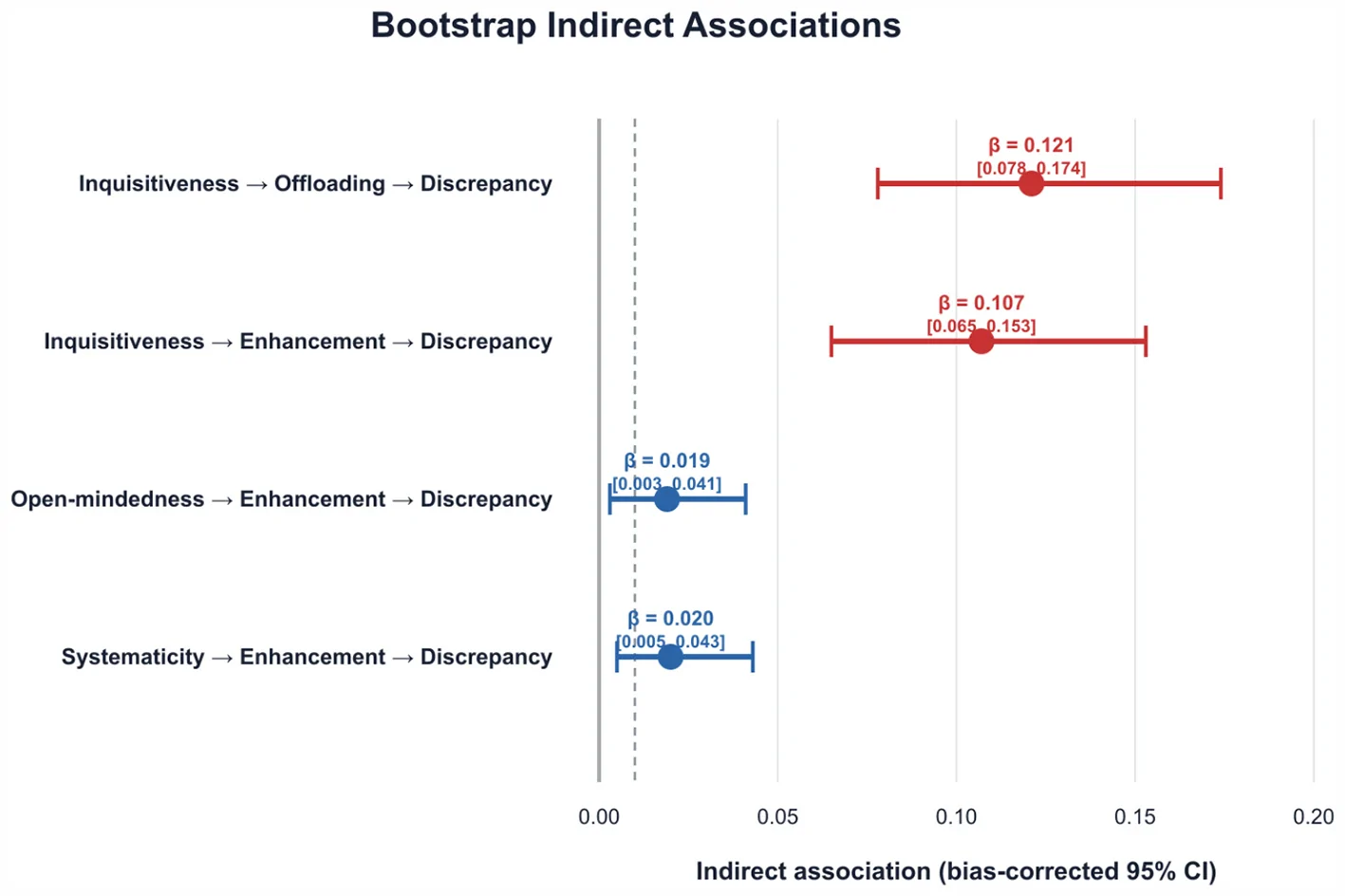
Forest plot of bootstrap indirect associations with perceived support-dependence discrepancy.

### 4.5 Covariate robustness

Gender, grade level, institution type, disciplinary background, and GenAI-use frequency were included as covariates in an auxiliary robustness model. GenAI-use frequency showed small positive associations with cognitive offloading, cognitive enhancement, and perceived support-dependence discrepancy, while most other covariate coefficients were small and non-significant. After adjustment, the direction and significance classification of H1a, H2a, H2d, H2e, H3, and H4 remained unchanged, and the absolute changes in the focal standardized coefficients were below 0.03. Complete covariate coefficients, standard errors, 95% confidence intervals, and *p* values are reported in Supplementary Table S4.

## 5 Discussion

This study estimated differentiated associations between five critical thinking dispositions, two GenAI interaction pathways, and perceived support-dependence discrepancy. The findings are consistent with several propositions derived from distributed cognition and depth-of-processing perspectives, while the cross-sectional and self-report design limits causal and mechanistic interpretation.

### 5.1 Heterogeneous associations between cognitive regulation and higher-order thinking in human–AI collaborative learning

Inquisitiveness showed the clearest dual pattern because it was associated with both cognitive enhancement and cognitive offloading, and both indirect associations were statistically distinguishable from zero. This result should not be read as evidence that inquisitive learners are dispositionally prone to uncritical dependence on GenAI. A more defensible interpretation is that stronger inquiry motivation may lead learners to pursue broader, more complex, and less clearly bounded questions in GenAI-supported learning environments. Under such conditions, exploratory engagement can coexist with partial delegation: learners may ask for alternative explanations and counterarguments, but they may also externalize preliminary search, organization, and response structuring when the scope of inquiry exceeds their available resources. The positive inquisitiveness–offloading association therefore reflects a high-engagement pattern that may include both elaboration and delegation, rather than a simple contradiction between curiosity and dependence. Longitudinal prompt traces, standardized task designs, and process-level indicators are needed to determine whether such delegation is strategic, habitual, or task-contingent.

Analyticity and truth-seeking did not show the expected associations with either interaction pathway. One possible explanation is that these dispositions influence behavior only when task cues prompt epistemic checking of external claims and metacognitive monitoring of one's own understanding. Fluent and internally coherent output may reduce perceived error risk and the subjective cues for both processes, even when generated content contains unsupported claims (Farquhar et al., 2024; Hoff and Bashir, 2015). This account is consistent with the metacognitive demands identified in GenAI interaction (Tankelevitch et al., 2024), but it remains inferential because neither epistemic checking nor metacognitive monitoring was measured as a separate process. Future studies should combine verification clicks, source-check records, prompt revisions, confidence judgments, and think-aloud data to test whether epistemic checking and metacognitive monitoring operate as distinct processes.

Open-mindedness and systematicity showed statistically significant but modest associations with cognitive enhancement. The small coefficients should not be characterized as strong educational effects. They are consistent with theoretical accounts in which open-minded thinking supports consideration of competing explanations and systematicity supports the organization of evidence and conclusions (Facione et al., 1995; Stanovich and West, 1997). Recent AI-supported learning studies likewise emphasize structured comparison, argument reconstruction, and scaffolded critical engagement rather than isolated acceptance of generated answers (Yan et al., 2025; Yusuf et al., 2024). Replication with independently validated GenAI-interaction scales and behavioral measures is nevertheless required.

### 5.2 Perceived support-dependence discrepancy and its theoretical interpretation

Both interaction pathways were positively associated with perceived support-dependence discrepancy. The stronger coefficient for offloading is consistent with the possibility that substitutive use of GenAI reduces opportunities for independent practice and makes unsupported task completion more difficult when AI support is removed. Importantly, this pattern should not be interpreted as evidence that offloading necessarily produces blind confidence. Given the wording of the outcome item, a high PSD score indicates that learners recognize a gap between AI-supported performance and unaided competence. The smaller positive coefficient for enhancement may reflect a different process: learners who actively question, compare, verify, and reconstruct GenAI outputs may become more aware of what they can and cannot reproduce independently. Thus, the positive associations observed for both pathways are more appropriately interpreted as perceived support-dependent competence gaps than as objectively verified overconfidence, learning loss, or calibration error.

A distributed-cognition perspective remains useful, but its role in this study is interpretive rather than evidential. GenAI-supported products are produced by a learner–tool system, and learners may subsequently evaluate whether the relevant reasoning, organization, or problem-solving steps can be reproduced without external support (Clark, 2025; Hollan et al., 2000). The present item captures this perceived supported-versus-unsupported contrast. It does not establish that learners confused their own contribution with system support, nor does it identify cognitive ownership as a mediator. Future studies should directly compare AI-assisted performance, unaided performance, and confidence judgments to determine whether support-dependence awareness, overconfidence, or calibration bias is present.

The Academic Stress × Cognitive Offloading interaction was not statistically significant, and the interaction added only Δ*R*^2^ = 0.002. These results indicate that the hypothesized moderation was not detected in the present observed-score model. They do not demonstrate that the offloading–support-dependence-discrepancy association is invariant across all stress levels, institutions, or learning tasks. The single-item stress indicator and heterogeneous task contexts may have reduced sensitivity to conditional effects.

The auxiliary covariate model did not materially change the direction or significance classification of the focal paths. GenAI-use frequency showed small positive associations with several endogenous variables, whereas most demographic covariates were small and non-significant. These results support the robustness of the focal associations within the present sample but do not establish that demographic or experiential factors are irrelevant in other populations or task contexts.

### 5.3 Indirect associations and support-dependence awareness

The significant indirect associations from inquisitiveness can be interpreted in different ways. The offloading pathway suggests that learners with stronger inquisitiveness may engage GenAI more broadly and, in some cases, delegate organization or reasoning steps, which is associated with a larger perceived gap between supported and independent task completion. The enhancement pathway suggests that active comparison, verification, and reconstruction may increase learners' awareness of the limits of their unaided competence. From a learning perspective, immediate and persistent AI assistance may reduce the desirable difficulties that normally reveal what has been independently encoded and may also weaken the diagnostic value of scaffold withdrawal (Bjork and Bjork, 2011; van de Pol et al., 2010). These interpretations remain provisional: the bootstrap analysis did not establish temporal order, and the study did not directly measure unaided performance, confidence calibration, or cognitive ownership. Experimental designs comparing AI-assisted performance, confidence judgments, and delayed unaided transfer are needed to evaluate these accounts directly.

### 5.4 Educational implications for higher-order thinking and metacognitive monitoring

The observed associations suggest that instructional design should distinguish temporary AI-supported performance from independently retrievable knowledge, while avoiding claims that the present survey establishes intervention effects. Inquisitiveness was associated with both cognitive enhancement and cognitive offloading, indicating that extensive exploratory engagement may combine constructive elaboration with partial delegation. The smaller positive associations of open-mindedness and systematicity with cognitive enhancement are consistent with, but do not demonstrate the effectiveness of, activities involving comparison and organized synthesis. Because both interaction pathways were positively associated with perceived support-dependence discrepancy, instruction may usefully make the boundary between AI-enabled task completion and unaided competence more visible. On this basis, structured reconstruction, metacognitive monitoring, and epistemic responsibility can be treated as a three-component, theory-informed design framework that remains to be evaluated experimentally (Molenaar, 2022; van de Pol et al., 2010; Yusuf et al., 2024).

Within this framework, source verification refers to the deliberate checking of an AI-generated claim against identifiable evidence, including the credibility, relevance, and consistency of the cited or independently located sources (Facione, 2000; Pennycook and Rand, 2019; Tankelevitch et al., 2024). Structured reconstruction refers to selecting verified material and reorganizing it within a learner-generated conceptual structure rather than reproducing the system's wording, sequence, or conclusion; this proposal is consistent with constructive and interactive engagement and scaffolded learning (Chi and Wylie, 2014; van de Pol et al., 2010; Yusuf et al., 2024). For example, in an evidence-based essay assignment, students could compare two GenAI responses, mark each substantive claim, link it to a course reading or another traceable source, identify unsupported assumptions, and then construct an independently organized argument map before drafting the essay. Accordingly, source verification and structured reconstruction are proposed as theoretically informed instructional practices for supporting students' metacognitive monitoring. Their effectiveness should be examined in future classroom-based experimental or quasi-experimental studies.

A classroom sequence can make students' metacognitive monitoring more explicit while making their own contribution to the task more observable. Before using GenAI, students first produce an unaided outline or explanation and record their confidence in their understanding. During GenAI use, they compare alternative outputs, annotate discrepancies, verify claims against course materials or other traceable sources, and document which ideas they accept, revise, or reject. After GenAI access is withdrawn, they complete a delayed unaided explanation or transfer task and compare it with their AI-assisted product. By comparing their initial confidence, AI-assisted work, and subsequent unaided performance, students can revise their judgments of what they understand and can reproduce independently. This sequence is proposed as a theoretically informed instructional practice for future classroom testing rather than as an intervention whose effectiveness has been established by the present cross-sectional study.

Epistemic responsibility can be operationalized through assessment criteria that award credit for traceable evidence, explicit reasons for accepting or rejecting AI-generated claims, documented revisions, and successful unaided transfer rather than polished output alone. Students can submit a concise audit trail identifying which components were adopted from GenAI, independently verified, substantially revised, or reconstructed without support. Because analyticity and truth-seeking were not significantly associated with either interaction pathway, verification should be explicitly scaffolded and assessed rather than assumed to follow from these dispositions. Likewise, because the Academic Stress × Cognitive Offloading interaction was not significant, the present findings do not justify stress-specific instructional prescriptions. Implementation should therefore be adapted cautiously to task demands, disciplinary context, permitted assistance, and students' patterns of GenAI use.

### 5.5 Limitations and future research

First, academic stress and perceived support-dependence discrepancy were measured with single items. Reliability sensitivity analyses showed that the focal latent-path conclusions were stable across several assumed reliability values, but this does not substitute for direct evidence of reliability, construct coverage, or measurement validity. The PSD item captures learners' perceived awareness of a gap between AI-supported and unaided competence; it does not directly measure the accuracy of ongoing judgments about understanding or independent reproducibility, nor does it establish illusory self-assessment, overconfidence, or objectively verified calibration bias. Future studies should develop and validate separate multi-item measures of perceived support-dependence discrepancy and metacognitive monitoring and combine them with repeated confidence judgments, AI-assisted performance scores, delayed unaided performance scores, and objective calibration indices.

Second, the cross-sectional design does not establish temporal or causal ordering. Structural paths and indirect associations should be interpreted as conditional covariance patterns under the specified model. Longitudinal panels, cross-lagged designs, randomized interventions, and delayed transfer tests are needed to evaluate directionality.

Third, convergent and discriminant validity were not uniformly strong. Three AVE values were below 0.50, and several critical-thinking dimensions showed high HTMT values and semantically overlapping items. Future measurement studies should revise overlapping items, confirm the factor structure in an independent sample, assess convergent and incremental validity against established AI-literacy and GenAI-use measures, and test measurement invariance across institutions and disciplines. Fourth, all focal variables came from the same questionnaire. Procedural controls and the common-method-factor analysis reduced but did not eliminate common-method concerns. Future studies should combine self-reports with prompt logs, source-check records, think-aloud protocols, instructor ratings, and objective transfer tasks to determine whether reported interaction patterns correspond to observable behavior. Fifth, participants answered with reference to usual academic tasks rather than a standardized task, so task type, complexity, discipline, and permitted assistance may be confounded. Future research should use standardized tasks and systematically vary or record task complexity, disciplinary context, and the permitted level of GenAI assistance to test whether the observed associations are task-contingent. Sixth, although the stratified purposive sampling strategy provided variation across regions and institution types, the sample was not probability-based. Future multicenter studies should adopt probability-based or clearly enumerated sampling frames, document institution-specific recruitment and exclusions, and test the model across a broader range of regions and institutions. The findings should therefore be generalized beyond the participating universities with caution.

## 6 Conclusion

This study examined the associations among five dimensions of critical thinking disposition, cognitive offloading, cognitive enhancement, and perceived support-dependence discrepancy in GenAI-assisted academic learning. Inquisitiveness was positively associated with both interaction pathways, whereas open-mindedness and systematicity showed small positive associations with cognitive enhancement. Analyticity and truth-seeking were not significantly associated with either cognitive offloading or cognitive enhancement. Both interaction pathways were positively associated with perceived support-dependence discrepancy, although the association was stronger for cognitive offloading.

The findings contribute to research on learner–AI interaction by distinguishing cognitively substitutive and cognitively constructive forms of GenAI engagement while recognizing that they may coexist. The significant indirect associations further indicate that critical thinking dispositions may be linked to perceived supported-versus-unsupported competence through different patterns of human–AI interaction. However, the study assessed perceived support-dependence discrepancy as a monitoring-related self-report rather than as a direct measure of metacognitive monitoring, cognitive ownership, or objective calibration. The present findings should therefore be interpreted as associations with a perceived support-dependent competence gap rather than as evidence of any single established psychological mechanism.

Academic stress did not significantly moderate the association between cognitive offloading and perceived support-dependence discrepancy in the present observed-score analysis. This result indicates only that the hypothesized moderation was not detected in the current sample and does not establish invariance across tasks, institutions, or stress conditions. Given the cross-sectional design, single-item outcome measure, common data source, and heterogeneous task contexts, the findings should be interpreted as evidence of conditional statistical associations. Longitudinal, behavioral, and experimental studies incorporating AI-assisted performance, unaided performance, confidence judgments, and validated support-dependence measures are needed to test the direction and mechanisms of these relationships.

## Data Availability

The original contributions presented in the study are included in the article/Supplementary material, further inquiries can be directed to the corresponding author.
