# Supplementary material for: Critical thinking dispositions and perceived support-dependence discrepancy in GenAI-assisted learning: evidence from human–AI interaction pathways
Source: Front Psychol. 2026 Oct 28;17:1913577. doi: 10.3389/fpsyg.2026.1913577 (PMC13752207; doi:10.3389/fpsyg.2026.1913577)
Supplement: Supplementary file 1 [file Supplementary_file_1.docx]

# Supplementary Materials

**Table S1. Alternative CFA Model Comparison**

| Model | χ² | df | χ²/df | CFI | TLI | RMSEA (90% CI) | SRMR | AIC | BIC |
| --- | --- | --- | --- | --- | --- | --- | --- | --- | --- |
| Seven correlated factors | 356.42 | 168 | 2.12 | .954 | .943 | .038 [.033, .043] | .035 | 482.42 | 777.31 |
| Higher-order CT | 397.85 | 173 | 2.30 | .945 | .935 | .040 [.035, .045] | .040 | 513.85 | 785.34 |
| Merged analyticity–truth-seeking–open-mindedness factor model | 561.73 | 176 | 3.19 | .907 | .890 | .052 [.047, .057] | .064 | 671.73 | 929.18 |
| Single factor | 1438.26 | 189 | 7.61 | .699 | .663 | .091 [.087, .096] | .102 | 1522.26 | 1718.86 |

*Note. The seven-correlated-factor model was retained for the focal analysis because it showed the best absolute fit and the lowest AIC and BIC. The acceptable higher-order model indicates substantial common variance among the five critical-thinking dimensions.*

Table S2. Complete HTMT Matrix and Key Bootstrap Confidence Intervals

| Construct | CO | CE | INQ | AN | TS | OM | SYS |
| --- | --- | --- | --- | --- | --- | --- | --- |
| CO | — |  |  |  |  |  |  |
| CE | .654 | — |  |  |  |  |  |
| INQ | .451 | .713 | — |  |  |  |  |
| AN | .354 | .449 | .675 | — |  |  |  |
| TS | .372 | .441 | .584 | .853 | — |  |  |
| OM | .347 | .428 | .503 | .781 | .861 | — |  |
| SYS | .339 | .426 | .509 | .824 | .801 | .887 | — |

**Key Bootstrap Confidence Intervals**

| Construct Pair | HTMT | 95% Bootstrap CI | Interpretation |
| --- | --- | --- | --- |
| Open-mindedness–Systematicity | .887 | [.851, .916] | High overlap; CI excludes 1.00 |
| Open-mindedness–Truth-seeking | .861 | [.823, .892] | Qualified separation |
| Analyticity–Truth-seeking | .853 | [.817, .884] | Above strict .85; below .90 |
| Cognitive Offloading–Cognitive Enhancement | .654 | [.610, .695] | Related but empirically distinguishable |

*Note. HTMT values were estimated for the seven multi-item constructs using 5,000 bootstrap resamples. None of the reported confidence intervals included 1.00. The combined evidence provides qualified rather than unequivocal support for discriminant validity.*

Table S3. Reliability Sensitivity for Perceived Support-Dependence Discrepancy

| Assumed Reliability | Offloading $\to$ Discrepancy $\beta$ | Enhancement $\to$ Discrepancy $\beta$ | $R^{2}$ Discrepancy |
| --- | --- | --- | --- |
| .70 | .489 | .226 | .365 |
| .80 | .461 | .213 | .347 |
| .85 | .448 | .207 | .340 |
| .90 | .437 | .201 | .333 |
| 1.00 | .418 | .192 | .319 |

Note. Perceived support-dependence discrepancy was modeled as a single-indicator construct. Its residual variance was fixed as (1 − ρ)sᵧ². Across assumed reliabilities from .70 to 1.00, the directions and significance classifications of the offloading and enhancement paths remained unchanged. The analysis should therefore be interpreted as a robustness check for the specified SEM paths rather than as evidence of full construct validity or objective measures of unaided performance.

Table S4. Complete Covariate Robustness Coefficients

| Covariate | Outcome | Std. β | S.E. | 95% CI | p |
| --- | --- | --- | --- | --- | --- |
| Gender (female = 1) | Cognitive Offloading | −.038 | .033 | [−.102, .026] | .248 |
| Gender (female = 1) | Cognitive Enhancement | .029 | .033 | [−.035, .093] | .374 |
| Gender (female = 1) | PSD (Support-Dependence) | −.021 | .032 | [−.084, .042] | .512 |
| Grade level | Cognitive Offloading | −.051 | .032 | [−.114, .012] | .112 |
| Grade level | Cognitive Enhancement | .058 | .032 | [−.005, .121] | .071 |
| Grade level | PSD (Support-Dependence) | −.042 | .032 | [−.104, .020] | .184 |
| Double First-Class institution | Cognitive Offloading | −.027 | .032 | [−.091, .037] | .405 |
| Double First-Class institution | Cognitive Enhancement | .046 | .032 | [−.018, .110] | .156 |
| Double First-Class institution | PSD (Support-Dependence) | −.023 | .032 | [−.086, .040] | .471 |
| STEM | Cognitive Offloading | .033 | .033 | [−.032, .098] | .321 |
| STEM | Cognitive Enhancement | .041 | .033 | [−.024, .106] | .218 |
| STEM | PSD (Support-Dependence) | .012 | .032 | [−.051, .075] | .709 |
| Arts and Sports | Cognitive Offloading | .047 | .034 | [−.019, .113] | .164 |
| Arts and Sports | Cognitive Enhancement | −.036 | .034 | [−.102, .030] | .284 |
| Arts and Sports | PSD (Support-Dependence) | .029 | .033 | [−.036, .094] | .379 |
| Computer Science and AI | Cognitive Offloading | .069 | .034 | [.003, .135] | .041 |
| Computer Science and AI | Cognitive Enhancement | .078 | .033 | [.013, .143] | .019 |
| Computer Science and AI | PSD (Support-Dependence) | .045 | .032 | [−.018, .108] | .162 |
| Agriculture, Forestry, and Medicine | Cognitive Offloading | .021 | .034 | [−.046, .088] | .541 |
| Agriculture, Forestry, and Medicine | Cognitive Enhancement | .034 | .034 | [−.032, .100] | .314 |
| Agriculture, Forestry, and Medicine | PSD (Support-Dependence) | .017 | .033 | [−.049, .083] | .611 |
| GenAI-use frequency | Cognitive Offloading | .128 | .039 | [.052, .204] | < .001 |
| GenAI-use frequency | Cognitive Enhancement | .146 | .044 | [.059, .233] | < .001 |
| GenAI-use frequency | PSD (Support-Dependence) | .073 | .032 | [.011, .135] | .021 |

*Note*. Humanities and social sciences served as the reference category for disciplinary dummy variables. Standard errors and confidence intervals are reported on the standardized scale. The direction and significance classification of the focal paths remained unchanged after adjustment, and absolute changes in focal standardized coefficients were below .03.

## Supplementary Appendix A. Questionnaire Reporting Version, Screening Questions, Attention Checks, Response Scales, and Variable Coding

The materials below constitute the English reporting version reconstructed from the analysis archive and manuscript documentation. They are provided for methodological transparency and should not be described as a verbatim export of the administered WJX questionnaire unless the original platform file is recovered and verified.

### A1. Study Information and Consent

Generative artificial intelligence (GenAI) refers to systems that generate text, explanations, plans, or answers in response to user instructions, such as ChatGPT, DeepSeek, Kimi, Doubao, ERNIE Bot, or Tongyi Qianwen.

Please answer with reference to your usual use of GenAI in university coursework, assignments, academic writing, information retrieval, and problem solving. Do not answer with reference to entertainment or other non-academic uses. Participation is voluntary and anonymous. You may discontinue before submitting the questionnaire. Submission of the completed questionnaire indicates informed consent. C1. After reading the information above, do you voluntarily agree to participate? Yes / No. Eligibility rule: Yes.

### A2. Eligibility Screening

S1. Are you currently enrolled as a university student? Yes / No. Eligibility rule: Yes.

S2. Have you used a generative artificial intelligence system for coursework, assignments, academic writing, information retrieval, problem solving, or another academic learning activity? Yes / No. Eligibility rule: Yes.

### A3. Demographic and Use Variables

D1. Gender: Male / Female. D2. Current study stage: Lower-division undergraduate (first-/second-year) / Upper-division undergraduate (third-/fourth-year) / Postgraduate. D3. Institution type: Regular university / Double First-Class university. D4. Disciplinary background: STEM / Humanities and social sciences / Arts and sports / Computer science and artificial intelligence / Agriculture, forestry, and medicine. D5. Frequency of GenAI use for academic learning: Less than once per month / 1–3 times per month / 1–2 times per week / 3–6 times per week / Daily or almost daily.

### A4. Response Scale for Multi-Item Constructs

For CO1–SYS3, use: 1 = strongly disagree, 2 = disagree, 3 = neither agree nor disagree, 4 = agree, 5 = strongly agree.

### A5. Cognitive Offloading

CO1. If the AI response meets the basic requirements of the assignment, I adopt it directly without further modification. CO2. I rely on GenAI to complete reasoning steps that I would otherwise be expected to perform independently. CO3. When a task is difficult, I allow GenAI to organize the main answer rather than independently constructing the response structure.

### A6. Cognitive Enhancement

CE1. I frequently input my own perspectives into the AI and ask it to generate counterarguments or identify logical flaws in my reasoning. CE2. Before deciding which argument or explanation is most convincing, I compare multiple explanations generated by GenAI. CE3. I revise and reorganize information provided by GenAI to form my own conceptual understanding.

### A7. Inquisitiveness

INQ1. When faced with a complex problem, I have a strong desire to explore the mechanisms underlying it. INQ2. I enjoy continuing to explore an unfamiliar issue even when doing so is not necessary to complete the immediate task. INQ3. When I encounter an explanation that I do not understand, I continue searching until I understand the underlying reason.

### A8. Analyticity

AN1. When reading a lengthy text, I actively look for logical leaps or flawed causal reasoning. AN2. I check whether conclusions follow logically from the evidence presented. AN3. When evaluating an explanation or argument, I try to identify its underlying assumptions.

### A9. Truth-Seeking

TS1. Before citing data or literature provided by the Internet or AI, I verify the original source. TS2. When reliable evidence conflicts with an existing conclusion, I am willing to revise that conclusion. TS3. I value accurate evidence more than information that merely supports my existing view.

### A10. Open-Mindedness

OM1. When conclusive evidence or AI reasoning contradicts my views, I rationally revise my perspective. OM2. Before deciding that my initial view is correct, I carefully consider alternative explanations. OM3. I can accept a well-supported argument even when it comes from a position different from my own.

### A11. Systematicity

SYS1. When confronted with fragmented information, I reorganize it to construct a clear framework. SYS2. I approach complex learning tasks through orderly steps. SYS3. Before reaching a final judgment, I organize the evidence and conclusions.

### A12. Academic Stress and Perceived Support-Dependence Discrepancy

AS1. Overall, how would you rate your current level of academic stress? 1 = very low, 2 = low, 3 = moderate, 4 = high, 5 = very high. PSD1. When I use AI to solve problems, I often feel that my ability to solve problems independently has improved; however, when I face similar tasks without AI, I find that I still cannot complete them independently. 1 = strongly disagree to 5 = strongly agree.

### A13. Instructional Manipulation Checks

IMC1. To show that you are reading the items carefully, please select “agree” for this item. Correct response: 4. IMC2. To support data quality, please select “strongly disagree” for this item. Correct response: 1.

### A14. Variable Coding

Gender: male = 0, female = 1.

Grade level: lower-division undergraduate = 1, upper-division undergraduate = 2, postgraduate = 3.

Institution type: regular university = 0, Double First-Class university = 1.

Disciplinary background: dummy-coded with humanities and social sciences as the reference category.

GenAI-use frequency: 1 = less than monthly, 2 = 1–3 times monthly, 3 = 1–2 times weekly, 4 = 3–6 times weekly, 5 = daily or almost daily.

CO, CE, INQ, AN, TS, OM, and SYS composite scores: arithmetic mean of the three corresponding items.

Both instructional manipulation checks had to be answered correctly for a submission to be retained. The archived analysis file preserved the final data-quality inclusion or exclusion flag but did not preserve the exact algorithmic threshold originally used to identify straight-line or otherwise patterned responding. This reflects a data-retention limitation in the archived analysis materials. Therefore, no unverified threshold or mutually exclusive reason-specific exclusion counts were reconstructed for this reporting appendix.
